# Characterizing chromatin landscape from aggregate and single-cell genomic assays using flexible duration modeling

**DOI:** 10.1038/s41467-020-14497-5

**Published:** 2020-02-06

**Authors:** Mariano I. Gabitto, Anders Rasmussen, Orly Wapinski, Kathryn Allaway, Nicholas Carriero, Gordon J. Fishell, Richard Bonneau

**Affiliations:** 1 Center for Computational Biology, Flatiron Institute, Simons Foundation, New York, NY 10010 USA; 2 New York University, Neuroscience Institute and the Department of Neuroscience and Physiology, Smilow Research Center, New York, NY 10016 USA; 3 Department of Neurobiology, Harvard Medical School, Boston, MA 02115 USA; 4 Stanley Center at the Broad, Cambridge, MA 02142 USA; 5 Scientific Computing Core, Flatiron Institute, Simons Foundation, New York, NY 10010 USA; 6 New York University, Center for Data Science, New York, NY 10010 USA; 7 New York University, Department of Biology, New York, NY 10012 USA

**Keywords:** Genomics, Statistical methods, Next-generation sequencing, Chromatin

## Abstract

ATAC-seq has become a leading technology for probing the chromatin landscape of single and aggregated cells. Distilling functional regions from ATAC-seq presents diverse analysis challenges. Methods commonly used to analyze chromatin accessibility datasets are adapted from algorithms designed to process different experimental technologies, disregarding the statistical and biological differences intrinsic to the ATAC-seq technology. Here, we present a Bayesian statistical approach that uses latent space models to better model accessible regions, termed ChromA. ChromA annotates chromatin landscape by integrating information from replicates, producing a consensus de-noised annotation of chromatin accessibility. ChromA can analyze single cell ATAC-seq data, correcting many biases generated by the sparse sampling inherent in single cell technologies. We validate ChromA on multiple technologies and biological systems, including mouse and human immune cells, establishing ChromA as a top performing general platform for mapping the chromatin landscape in different cellular populations from diverse experimental designs.

## Abstract (web summary)

Most currently available statistical tools for the analysis of ATAC-seq data were repurposed from tools developed for other functional genomics data (e.g. ChIP-seq). Here, Gabitto et al develop ChromA, a Bayesian statistical approach for the analysis of both bulk and single-cell ATAC-seq data.

## Introduction

The genome of eukaryotic cells is tightly packed into chromatin^1^ with only a fraction of chromosomal regions accessible within any given cell population at a particular developmental stage. Chromosomal accessibility plays a central role in several nuclear processes, including the regulation of gene expression and the structure and organization of the nucleus^2^. Chromatin remodelers modify chromatin state creating structural changes that affect gene expression^3,4^. Transcription factor proteins (TFs) are key transcriptional regulators and chromatin remodelers, binding to accessible DNA regions to control the expression of genes^5^ and to inaccessible chromatin, altering the accessibility of targeted regions^6^. Differential expression and regulation of TFs act as a combinatorial code that gives rise to the wide repertoire of cellular phenotypes observed in mammalian organisms^7,8^.

The development of high-throughput chromatin accessibility assays (e.g., ATAC-seq) has enabled the analysis of chromatin accessible regions, the discovery of nucleosome positions and the characterization of transcription factor occupancy with almost single base-pair resolution^9^. In part due to the small initial starting material required (on the order of 10,000 cells) and from a desire to query the chromatin structure of particular rare cellular types, ATAC-seq has become widely adopted. Recent advances have improved the technique and enabled the mapping of the accessible chromatin landscape of individual cells^10^. Following the same trend, low starting material techniques to probe the methylome landscape and different chromatin features have evolved from bulk assays to the single-cell domain^11–13^. These techniques raise the possibility of both describing the variability of chromatin accessibility, methylation states and chromatin fragments, and enable the study of epigenomic heterogeneity by classifying cellular types based on their chromatin structure^13–15^.

Here, we present ChromA, a Bayesian statistical approach to characterize the chromatin landscape of aggregated and single cells and apply the method to multiple experimental technologies. In the case of ATAC-seq experiments, ChromA infers chromatin accessibility landscape and annotates accessible and inaccessible chromatin regions. ChromA harnesses recent developments in hidden semi-Markov models (HSMM) to create a scalable statistical inference method that can be applied to genome-wide experiments^16^. ChromA is able to integrate information from different experiments, and draw statistical power to create consensus chromatin annotations. To validate our method, we use Th17 bulk^17,18^, A20 and GM12878 single-cell data sets (the Data availability section), identifying accessible chromatin and establishing ChromA as an effective platform for mapping the chromatin landscape in different cellular populations. We show that the method is readily adaptable to different experimental designs and technologies.

## Results

### A hidden semi-Markov model for chromatin accessibility annotation

**Fig. 1:**
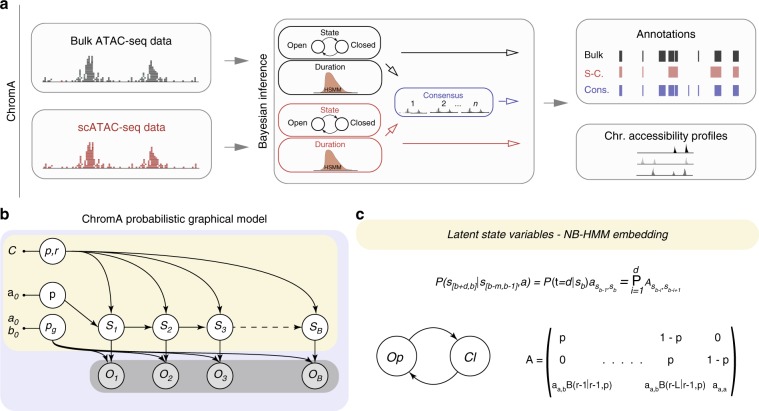
Overview of chromatin accessibility annotation algorithm. **a** ChromA is an easy to use algorithm that combines single and multiple BAM files (raw reads) or TSV files (list of Tn5-binding events) to create chromatin accessibility annotations. **b** Probabilistic graphical model describing ChromA’s structure. In this representation, nodes describe random variables and arrows depict dependencies among the variables. ChromA models the number of Tn5-binding events observed at each base using observed variables **O** (representing the number of binding event), and latent variables **S** (representing chromatin state). Subscripts denote base position, ranging from 1 to the length of a chromosome, *B*. Observed variables **O** are modeled using a geometric distribution with parameter *p*_*g*_. Chromatin-state variables **S** are subjected to semi-Markovian dynamics, depending on the previous chromatin state. *π* described the initial chromatin state, and *p* and *r* characterize the semi-Markovian transition matrix dictating chromatin-state context. **c** Our ATAC-seq pipeline using bulk measurements annotates chromatin using two states, open Op or closed Cl. Both states are characterized by semi-Markovian dynamics. The probability of annotating chromatin in bases *b* to *b* *+* *d* given previous chromatin states depends on two factors: the probability of transitioning between states, symbolized by transition matrix *a*, and the probability of dwelling in the new states during *d* bases. When the duration is characterized by a NB distribution with parameters *p* and *r*, the transition matrix can be re-written using an embedding matrix *A*. In the figure, we reproduce a simple transition matrix *A*, in which *p* and *r* are the NB parameters, *a* is the transition matrix between states, and *B* represents the binomial coefficient.

ChromA is a probabilistic graphical model developed to annotate chromatin regions as open (accessible) or closed (inaccessible) when experiments are performed on pooled (bulk), single cells, or a combination of both bulk samples and single cells. We first describe the results aimed at delineating accessible regions, and then illustrate extensions of the method to other tasks and technologies. Our algorithm takes as an input ATAC-seq-aligned sequencing reads (.bam files) or locations of Tn5-binding events (.tsv files) and produces chromatin accessibility annotations and quality control metrics for the data set (Fig. 1a).

ChromA is based on a Bayesian statistical model that encompasses a set of latent variables (**S**) representing chromatin states (namely chromatin accessibility) at each base (*b*) and a set of observations (**O**) composed by the reads (Fig. 1b). In our chromatin accessibility model, the chromatin state of each base is a binary variable representing two chromatin configurations, open (*S*_*b* _= 1) and closed (*S*_*b*_ = 0). Bayesian inference creates posterior estimates of model’s parameters by combining our prior belief about parameter values with the likelihood of the observations being generated by the model. In our case, ChromA aims to estimate posterior chromatin state by combining our prior belief on the accessibility of each base with the likelihood of generating the observed reads.

To model the duration of accessible regions from ATAC-seq experiments, we reason that contextual information plays a key role in defining each base’s annotation. To improve upon the duration behavior of standard hidden Markov models (HMM)^19^, we model the duration (*d*) of each accessible region through an HSMM that exhibits a flexible negative binomial (NB) duration distribution^20^ as follows.

1 $$\begin{array}{l}d\sim NB\left( {d;r,p} \right) \Leftrightarrow P\left( {d = l + 1{\mathrm{|}}r,p} \right)\\ = \left( {\begin{array}{*{20}{c}} {l + r - 1} \\ l \end{array}} \right)p^r(1 - p)^l\end{array}$$

The NB distribution has two parameters: an integer parameter *r* *>* 0, and a probability parameter 0 *<* *p* *<* 1. We use this distribution to capture the notion that cis-regulatory transcriptional machineries, necessary for accessing DNA-binding domains, might occupy a certain characteristic length. This length is in turn reflected in the size of chromatin accessible regions. The maximum or mode of a NB distribution is given by its parameters (mode = $\frac{{p(r - 1)}}{{(1 - p)}}$). This is contrary to models based on the geometric distribution (like previous HMMs) for which the maximum is fixed and always reached at 1 (Supplementary Fig. 1a).

Recent developments in approximate posterior calculation provide efficient techniques for the estimation of HSMM parameters. These techniques are advantageous when the duration of HSMM states are distributed according to a NB distribution^16^. To harness the advantage of such developments, we focus on the parameter that encodes the duration of each state in HMMs and HSMMs: the transition matrix. The transition matrix of a HSMM, $A\prime _{i,j}$, under the assumption of independence on the previous state duration, can be written using two terms: the probability of transitioning into a new state (*j*) from a current state (*i*), *A*_*i,j*_, and the probability of dwelling in the new state for a duration of d bases, *P*(*τ* = *d*|*S*_*b*_ = *j*) as follows.

2 $$P\left( {S_{\left[ {b,b + d} \right]} = j{\mathrm{|}}S_{b - 1} = i} \right) = A^\prime _{i,j} = P\left( {\tau = d{\mathrm{|}}S_b = j} \right)A_{i,j}$$

To facilitate inference, we begin by re-writing the NB distribution as a sum of shifted geometric distributions

3 $$\begin{array}{l}d\sim NB\left( {d;r,p} \right) \Leftrightarrow d = 1 + \mathop {\sum}\nolimits_{i = 1}^r {z_i} ,\\ {\mathrm{where}}\quad z_i\sim ShiftedGeo(1 - p)\end{array}$$

where the probability mass function of a *ShiftedGeo*(1 – *p*) is *p*(*z*|*p*) = *p*^*z*^(1 – *p*) with *z* an integer *z* ≥ 0. Equality 5 permits to write an HSMM’s transition matrix with NB distributed states, establishing a correspondence to a transition matrix, in which each state solely dependent on the previous one (HMM) (Fig. 1c). The new formulation creates an HMM embedding of a HSMM. An HMM embedding permits the use of inference machinery developed for the estimation of parameters in HMMs with a computational complexity that scales as O(*r*) for each state.

Next, we model the data-generating distribution that represents the likelihood that reads in a certain genomic region are generated by open or closed chromatin. The core element of the ATAC-seq assay is a modified version of the Tn5 transposon^9^. After preferential binding to accessible DNA, Tn5 transposase tagments DNA, leaving behind a DNA adaptor. A correctly oriented second event can be used to sequence the intervening fragment and to identify tagmented locations^21^. However, we lack information about the total number of cells in the assay and the maximum number of binding events available to each cell on a base pair per chromosome level. Due to the sparse nature of each binding event (especially in the case of small sample size and single-cell data sets, Supplementary Fig. 1a), we observed that a geometric distribution effectively represents the number of events present at each base of open and closed chromatin, completely specifying our initial Bayesian approach. In summary, the presented probabilistic graphical model provides predictive insight into chromatin state and as such defines its accessibility.

### Validating chromatin accessibility annotations

**Fig. 2:**
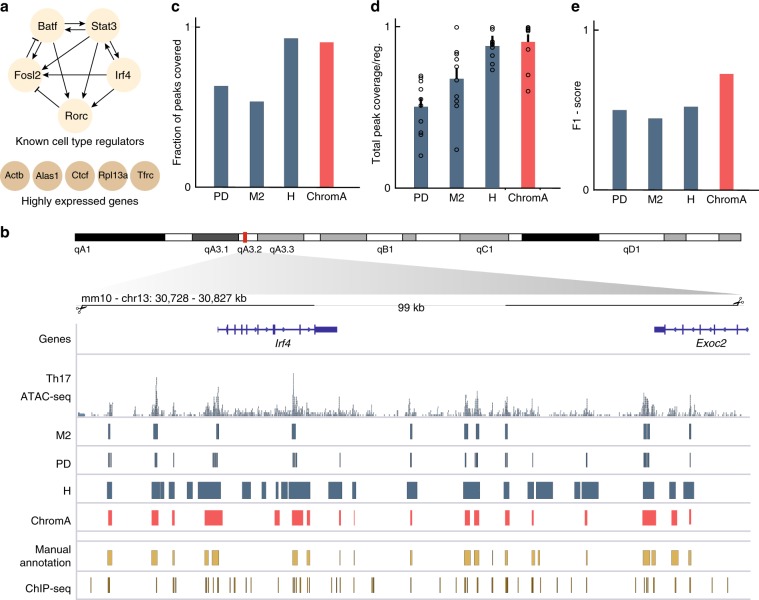
Validation of ChromA on ground-truth data sets. **a** Genomic loci were selected to create a validation data set from highly expressed and transcription factor genes regulating Th17 development. To manually curate genomic regions, information from ChIP-seq and ATAC-seq experiments were combined. **b** Example of curated genomic locus flanking the *Irf4* gene in the mouse genome. ATAC-seq, ChIP-seq, PeaKDEck (PD), MACS2 (M2), ChromA, and manual annotations are displayed. ChromA algorithm recovers a greater number of ground-truth peaks than competing algorithms and covers each peak more thoroughly. **c** Fraction of manually annotated peaks covered with at least one peak. **d** Average fraction of peaks covered per genomic loci (mean +/− s.e.m., the number of regions equals 10). **e** F1 score.

We focused on validating our method on the data collected from Th17 cells for which a validated regulatory network delineating their differentiation has been identified^17,18^. ATAC-seq, several histone marks, and ChIP-seq on focal transcription factors all of which play a deterministic role in cell fate commitment have been assayed in FACS-sorted Th17 cells^17,18^. We combine this information and manually annotate ten well-studied loci for this cell type, each ~100 kb in size, consisting of regulatory regions surrounding highly expressed genes and master regulator TFs (Fig. 2a). We based our curated annotations on the integration of information from (i) the existence of ATAC-seq regions with higher number of binding events than background, (ii) the occurrence of H3K27 acetylation marks^22^, and (iii) the presence of an accumulation of ChIP-seq-binding events (Supplementary Fig. 2). Three experimental experts annotated each region and only fully concordant bases were taken as ground-truth values for comparison to evaluate our model’s performance. We use these annotations to illustrate model development and initial performance evaluation.

Next, to assay chromatin annotations (Fig. 2b), we use three different metrics: the fraction of the total number of manually annotated peaks that contains at least one peak generated by the algorithm under consideration (fraction of peaks covered), the average fraction of coverage of each peak (average peak coverage), and F1 score. We used these metrics to compare ChromA annotations against PeaKDeck^23^ and MACS2^24^, two of the most commonly used tools to annotate the ATAC-seq data. In addition, we contrast ChromA against a recently developed tool to annotate accessible chromatin based on HMMs, HMMRatac^25^. Against PeaKDeck and Macs2, ChromA annotations not only recovered a higher fraction of correctly annotated peaks but also on average generated better coverage of each of the accessible regions (Fig. 2c–e). In addition, ChromA’s annotations control false discovery rate, particularly noticeable when compared against HMMRAtac. ChromA creates each base’s annotations by inferring the expected posterior mean of chromatin state using approximate Bayesian inference (additional computational acceleration is achieved through biologically inspired approximations; Supplementary Note 2, Supplementary Figs. 3–4). Posterior chromatin state is then thresholded by a fixed value set a priori (during our previous computational experiments, we set the threshold to 0.05). This algorithmic parameter does not play a major factor in ChromA’s annotations, as the number of regions recovered remain constant throughout a wide threshold range, highlighting the robustness of our model (Supplementary Fig. 5).

**Fig. 3:**
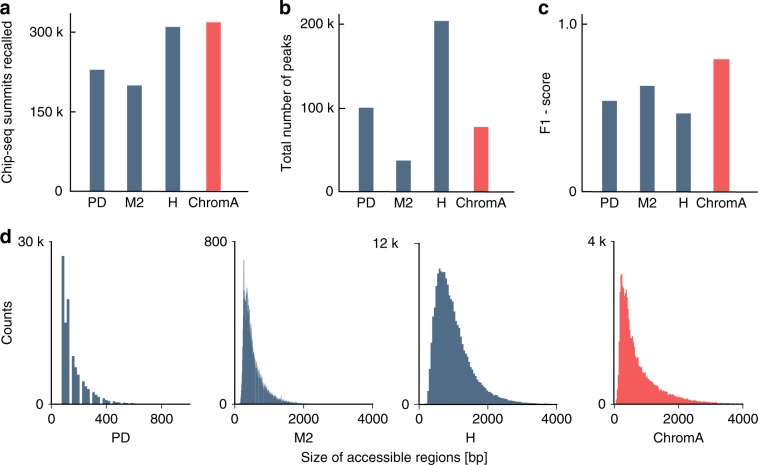
Genome-wide validation of chromatin accessibility annotations. **a**–**c** ChromA effective genome-wide performance recalls the highest number of ChIP-seq calls maintaining a comparable number of peaks and controlling false positives. **a** The number of ChIP-seq-binding events recalled. **b** The total number of peaks annotated. **c** F1 score computed as the harmonic mean between precision and recall. **d** Distribution of accessible regions for different chromatin annotation algorithms. Histograms depicting the size of accessible chromatin regions annotated by PeaKDEck, MACS2, HMMRAtac, and ChromA. Posterior size distribution of ChromA’s accessible regions resembles a NB distribution, also observed in MACS2 and HMMRAtac. PeaKDEck distribution is highly dependent on algorithmic parameters, such as window size, etc.

Next, we examine ChromA’s performance genome wide on Th17 cells. In this case, manual annotation is not feasible for computing a ground-truth metric (with changes in chromatin accessibility spanning the full genome^17,18^). Instead, we reasoned that ChIP-seq locations can be used as a proxy to indicate chromatin accessible regions and therefore used the ChIP-seq data for validation experiments. Compared with other existing methods, ChromA’s predictions faithfully recover the greatest number of ChIP-seq calls while maintaining a comparable total number of peaks while controlling false discovery rate (Fig. 3a, b). We summarize algorithmic performance using a precision recall curve and F1 score (see Supplementary Methods). ChromA outperforms competing approaches in these metrics (Fig. 3c; Supplementary Fig. 6). While, MACS2 and ChromA exhibit NB distributed sizes, PeaKDEck exhibits a discontinuous size distribution, in which an algorithmic parameter (peak size parameter) is a major determinant of its shape (Fig. 3d).

Lastly, we validated ChromA’s performance on four additional data sets, two of them consisting of Th17 cells and the remaining two consisting of CD4+ cells differentiated into Th17 cells (Supplementary Table 1). To differentiate CD4+ into Th17, CD4+sorted cells were purified by cell sorting and cultured for 48 h in Th17 differentiating media^17^. On these data sets, ChromA’s recovered on average 45% more peaks than MACS2, considering our genome-wide validation assays (Supplementary Fig. 6). Taken together, these results established ChromA as a top performing tool for discovering accessible chromatin regions from ATAC-seq data sets.

### Chromatin annotations from single-cell measurements

**Fig. 4:**
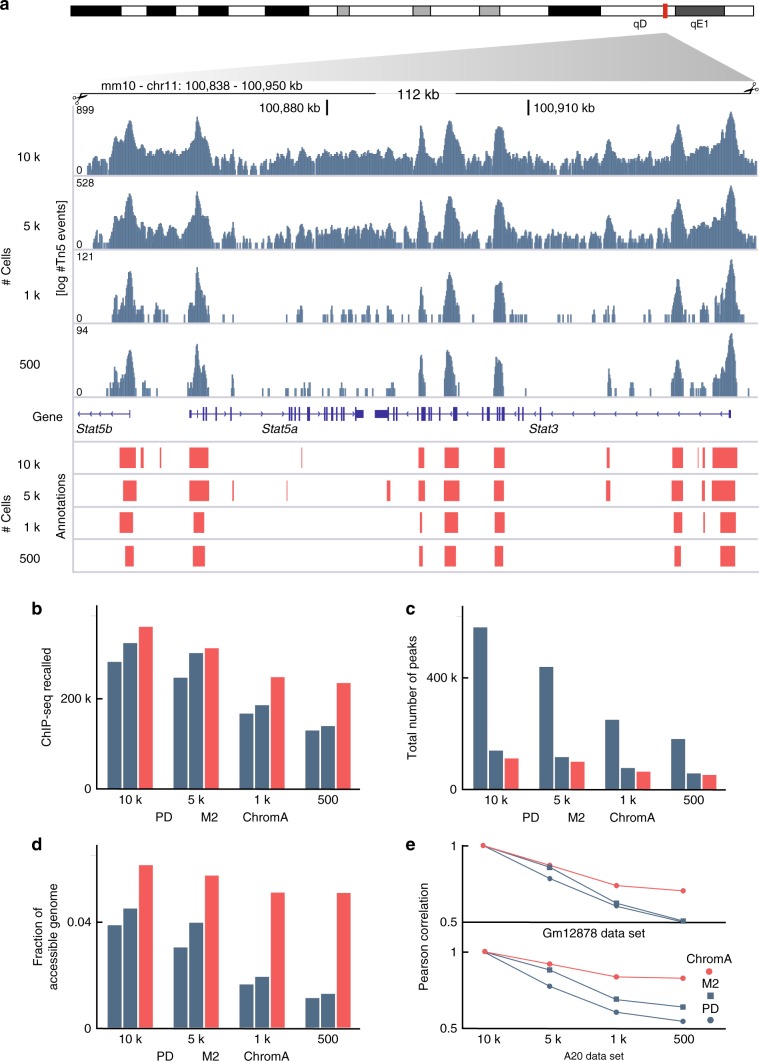
ChromA annotations generalize to single-cell data sets. **a** Annotations of mouse A20 single-cell data sets at the *Stat3* genomic locus. Cells are downsampled from 10,000 to 500 cells. ChromA annotations are consistent at different cell depths. **b**–**e** ChromA extends its effective genome-wide performance to single-cell data sets, again, recalling the highest number of ChIP-seq calls in GM12878 single-cell data sets. ChromA is particularly effective at low cell depths. **b** The number of ChIP-seq peaks recalled, **c** the total number of peaks and (**d**) fraction of the genome annotated as accessible for each downsampled data set. **e** Correlation between annotations at different cell depths calculated against the entire data set possessing 10,000 cells.

We extend ChromA’s core model beyond bulk processing to characterize chromatin accessibility in single cells (Fig. 4a). Here, we focus our analysis on single-cell data sets of mouse B lymphocyte A20 and human lymphoblastoid GM12878 cells (data set obtained from 10× Genomics, see Supplementary Methods section for a description of the samples). In addition to often-discussed sampling and technical issues, we find that single-cell ATAC-seq data sets also exhibit higher dynamic range (DR) than their bulk counterparts (bulk DR ~4 bits, single-cell DR ~11 bits; Supplementary Fig. 7). To characterize single-cell chromatin data sets and compare them to bulk data sets, we employ a set of metrics aimed to quantify data set quality. We compute a signal-to-noise ratio (SNR) centered around gene promoter regions, the fraction of reads in accessible regions, and a ratio between read lengths centered around mono-nucleosome and nucleosome-free regions (Supplementary Table 1, Supplementary Fig. 8).

To study the robustness of ChromA’s single-cell approach, we varied the total number of cells in our data sets and studied how chromatin annotations varied as we downsampled this single-cell data set data to different depths. ChromA’s annotations recovered the highest number of ChIP-seq calls and annotated the highest accessible genome fraction at every cell depth, consistent with ChIP-seq information (Fig. 4b–d). Taken together, our computational experiments validate our algorithms as an effective platform for chromatin annotation under different experimental settings.

### ChromA consensus integrates replicate information

**Fig. 5:**
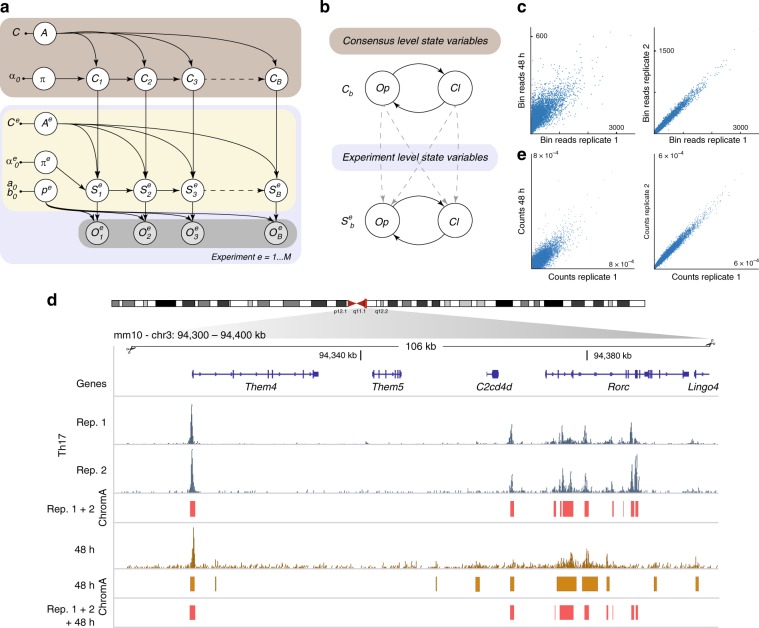
Consensus ChromA integrates information from different data sets and replicates. **a** Consensus ChromA probabilistic hierarchical graphical model. The model is divided into a top (light brown) and a bottom layer (light blue). The bottom layer shows ChromA’s probabilistic model for each data set analyzed (akin to Fig. 1b). The top layer schematic shows how consensus variables, **C**, are explicitly linked to latent-state variables for each replicate (or integrated experiment) according to Markovian dynamics. *π, p*_*g*_, and *A* variables are as in Fig. 1b. **b** Consensus and experiment variables *C* and *S*^*e*^ evolve alternating between open, Op, and closed, Cl, states. The top and the bottom layers are linked by introducing into each experiment a dependency on the state of the consensus variable. **c** Raw read correlation between replicates of sorted Th17 cells’ data sets (right) and Th17 cells against CD4+ cells incubated in Th17 differentiation media for 48 h (left). **d** Consensus-ChromA annotations integrates information from different replicates creating and deleting accessible regions based on the context. Raw reads from sorted Th17 cells’ replicates and sorted CD4+ cells at a genomic locus. ChromA annotations for single CD4+ data set. Consensus-ChromA annotations for Th17 replicates and Th17 replicates and CD4+ cells are shown in red. **e** Consensus ChromA creates a common representation of chromatin accessible regions. When both Th17 replicates are combined together with CD4+ cells, the resulting consensus representation maintains the high correlation observed only when both Th17 cells’ replicates are used. CD4 + peaks are filtered, and only correlated peaks survived.

We designed ChromA to infer a consensus chromatin-state representation by harnessing the statistical power from different experimental replicates, different clusters of cells, or sets of related experiments, thus inferring a more confident posterior estimate. In contrast to methods that select a repertoire of peaks from individual calculations on each replicate^26^, ChromA integrates information from different replicates on a base-by-base level. Our model consists of consensus and individual experiment chromatin-state variables (indicated with letter *C* and *S*^*e*^, respectively; Fig. 5a). We maintain NB HSMM dependencies in our consensus chromatin-state variable, *C*. Next, we formulate variables *S*^*e*^ such that they behave under semi-Markovian dynamics and incorporate a dependency on the state of the consensus representation (Fig. 5b). To model this dependency, we resort to the HMM NB embedding of the HSMM.

We augment individual experiment NB embedding to include a transition matrix, depending on the consensus representation. The link between each experiment and the consensus representation is possible because the HMM NB embedding, indicated with **C**, **S**^**e**^ below, creates a base-by-base dependency as follows.

4 $$P\left( {S_{\left[ {b,b + d} \right]}^e{\mathrm{|}}S_{b - 1}^e,C_{\left[ {b,b + d} \right]}} \right)= \left[ {P\left( {\tau = d{\mathrm{|}}S_b^e = j} \right)a_{i,j}} \right]P\left( {S_{\left[ {b,b + d} \right]}^e{\mathrm{|}}C_{\left[ {b,b + d} \right]}} \right)$$

where the letter *e* is an index for each experimental replicate. Equation (6) represents the HSMM probability of transitioning from a state at base *b* − 1 into a state spanning bases *b* to *b* + *d*, given consensus variables at those bases. This probability factorizes into a HSMM transition term times a term linking each experiment to the consensus variables. We re-write the previous equality by using the HMM NB-embedding transition matrix, *A*^*e*^, and a base-by-base consensus link transition matrix *H*.

5 $$P\left( {S_b^e = j{\mathrm{|}}S_{b - 1}^e = i,C_b = k} \right) = A_{i,j}^eH_{k,j}$$

To demonstrate the model’s efficacy in integrating information from replicate experiments, we apply this new statistical tool to an ATAC-seq data set comprised two biological replicates of Th17-sorted cells (Fig. 5d). The raw signals of replicates are highly correlated (correlation coefficient = 0.99, deepTools^27^). In addition, to study the model’s robustness to outliers, we select a lower correlation data set (CD4 + T cells cultured in Th17 conditions for 48 h, correlation coefficient = 0.68, Fig. 5c).

To assess our model’s performance, we measured the level of correlation among data sets based on the number of Tn5 transposition events occurring at each accessible chromatin region. In this case, replicates continue to be highly correlated, as expected (correlation coefficient = 0.99, accessible regions calculated with consensus-ChromA run only on Th17 cells replicate 1 and 2). This correlation remains unaltered even, when the outlier is included into the analysis (correlation coefficient = 0.99, accessible regions calculated with consensus-ChromA run on Th17 cells replicate 1, 2, and 48 h cultured CD4+; Fig. 5e). Although consensus ChromA builds accessible regions common to the three data sets, this common basis does not alter the fact that 48 -h cultured CD4+ cells correctly stand as an outlier, the individual model, S, for the 48 h cultured CD4+ cells is not perturbed (correlation coefficient replicate 1 vs 48 h = 0.651, correlation coefficient replicate 2 vs 48 h = 0.655; accessible regions calculated with consensus-ChromA run on Th17 cells replicate 1, 2, and 48 -h cultured CD4+).

A plethora of novel high-throughput technologies are emerging to characterize different layers of epigenomic regulation, including techniques that harness the ability of the Tn5 transposon to randomly integrate in the genome, nucleases, and other biomolecular methodologies^28–32^. A common first step shared among studies using these techniques is the identification of relevant regulatory regions, either in bulk or single-cell experiments. ChromA represents a general framework for the recognition of regulatory and functional regions that can be easily extended to annotate different experimental technologies. We adapt our algorithms to identify transcription factor-binding events from Cut&Run sequencing experiments^33^ and to annotate accessibility from DNAse-seq experiments (Supplementary Figs. 9, 10), illustrating a broad utility across diverse experimental designs and technologies.

## Discussion

A major goal in epigenomic analysis is to systematically characterize the different layers of epigenetic regulation in cell types at different developmental time points and under different conditions. To address these challenges, we developed ChromA, a powerful probabilistic model for the analysis of the unstructured epigenetic landscape, and demonstrated its ability to annotate chromatin accessible areas in the genome when tackling ATAC-seq experiments. We validated our approach with curated regions in the mouse genome and by assessing our algorithm performance against chromatin immunoprecipitation binding events, a proxy of accessible chromatin. We demonstrate that our probabilistic algorithm is useful both in single-cell and aggregate populations, being able to integrate information from replicates. These analyses show that our method can be readily extended to more complex models and experimental designs as new technologies emerge.

Our algorithm has several advantages over previous approaches. ChromA is the first algorithm to model entire genomes that handles state duration in a principled and data-driven manner, performing annotations at base-pair level via an explicit statistical model enabling variable-state length. A previous HSMM approach annotates small genomic segments via a windowing approach, limits state duration by a hard threshold, and it is not thoroughly validated (or validated on any of the newer genomic technologies described here)^34^. ChromA also improves over HMM-based algorithms^25,35,36^ by handling replicates and validating single-cell experimental designs. Some prior algorithms summarize genomic information in bins and are not designed to process ATAC-seq information. Semi-automatic segmentation methods, ChromHMM and Segway^37,38^, are also related to ChromA. ChromHMM is designed to process data sets using 200 bp bins. Segway introduces duration assumptions into its postulates, however, it does so by complex heuristics and many latent variables. Neither method is validated in the context of analysis of single data sets of any experimental technologies, such as ATAC-seq or Cut&Run. These methods are developed to aggregate information from a variety of different experimental assays, requiring user input to interpret their results. Finally, approaches modeling ATAC-seq information at the single-cell level^39–40^ or performing transcription factor footprinting^41,42^ are complementary to ChromA and benefit from a method that pre-selects relevant regulatory regions.

Focusing here on ATAC-seq experiments, ChromA exhibits several prominent features. First, by recovering wider accessible regions, ChromA captures valleys in read density associated with transcription factor footprinting. ChromA also exhibits higher sensitivity allowing for the recovery of less prominent peaks. As a result, single cells data sets, exhibiting an extended dynamic range compared with bulk measurements, can also be analyzed with our software. Finally, by integrating different experiments, ChromA is able to create a consensus annotation and thereby increase the signal-to-noise ratio (while still tolerating outlier regions or even mislabeled/outlier experiments/replicates). This analysis indicates that additional insights can be extracted by integrating different sources of information. In the future, we plan to extend ChromA to integrate different experimental procedures, extracting and combining information in a hierarchical fashion from a wide range of approaches.

## Methods

### Bulk ATAC-seq libraries and preprocessing

ATAC-seq libraries were downloaded from NCBI’s GEO Database under accession GSE113721. The following preprocessing pipeline was used to generate aligned reads. Adapters were trimmed using cutadapt. Reads were aligned using Bowtie2 to the murine mm10 reference genome and then filtered for mapping quality greater than Q30. Duplicates were removed using Picard (http://picard.sourceforge.net), and subsequently, mitochondrial, unmapped and chromosome Y reads were removed. For peak-calling, ChromA corrects the read start sites to represent the center of the tagmentation binding event, the +strand were offset b +4 bp, and all reads aligning to the—strand were offset −5 bp. In addition, ChromA filters peaks using a custom list that combines blacklisted genomic regions from the ENCODE project (http://mitra.stanford.edu/kundaje/akundaje/release/blacklists/mm10-mouse/mm10.blacklist.bed.gz). This filtering step takes place when building the set of transposition events by removing all the events falling into the blacklisted regions.

### Single-cell ATAC-seq Libraries

Single-cell data sets were downloaded from 10× genomics https://support.10xgenomics.com/single-cell-atac/datasets/1.0.0/atac_v1_hgmm_10k. Briefly, they consist of a mixture of fresh-frozen human (GM12878) and mouse (A20) cells collected with the Chromium Single Cell ATAC platform, and demultiplexed and pre-processed with the single-cell ATAC Cell Ranger platform. Cells were sequenced on Illumina NovaSeq with ~42 k read pairs per cell. Downsampled data sets are provided from the online website. TSV files are provided listing Tn5-binding events. ChromA incorporates the ability of importing TSV and Tabix files directly from Cell Ranger pipelines.

### Data set metrics

ChromA reports different quality control metrics to assess data set quality. Given ChromA annotations, the fraction of reads in peaks (FRIP) is calculated as the number of reads laying within peaks versus the total number of reads in chromosome 1. This is calculated using properly paired and mated reads. SNR is calculated by defining promoter regions in the mouse or human genome as regions spanning 1 kb upstream, 3 kb downstream from gene start sites. Insert size distribution is reported as an additional file, and insert size metric is computed as the ratio between the number of reads with insert size between 190 and 210 bp to the number of reads with insert size between 60 and 80 bp for chromosome 1. Finally, we extrapolate the number of properly paired and mated reads by computing that number for chromosome 1 and multiplying by the total length of the genome and then dividing by the length of chromosome 1.

### Detection of chromatin accessible regions

To perform experiments to validate our algorithm, we ran ChromA in each sample individually using standard priors (described below). An example of running ChromA on a wild-type data set of Th17 cells, using the mouse genome with our bulk model is detailed next: ChromA -i “Th17_1_noMito.bam” -species mouse -sb th17_wt1.bed. We ran PeaKDEck (parameters –bin 75, -STEP 25, -back 10000, -npBack100000). Peaks were identified using the MACS2 software. We run MACS2 using two sets of parameter, and always compare against the best-performing set (parameters: -m 10,30 -g 1865500000 -bw = 200 or -nomodel -shift -100 -extsize 200 -broad -keep-dup all).

### Transcription factor-binding prediction

TF ChIP-seq and control sequencing data were downloaded from GEO (GSE40918), mapped to the murine genome (mm10) with Bowtie2 (2.2.3), filtered based on mapping score (MAPQ > 30, SAMtools (0.1.19)), and duplicates removed (Picard). Peaks were identified using the MACS2 software (version 1.4.2) using the settings (parameters: -m 10,30 -g 1865500000 -bw = 200) and retained for raw *p*-value <10^−10^. All data sets were processed against an appropriate control. We retained summit locations to create a binding event localizing at a particular base pair.

Transcription factor-binding events for GM12878 were downloaded from ReMap^35^ (http://pedagogix-tagc.univ-mrs.fr/remap/celltype.php?CT=gm12878) by filtering the database for the cell type GM12878. There are 131 TFs in this database that correspond to the particular cell line, among which we can find CTCF, Pou factors, and members of the Pax, Stat, and Etv families.

### Validation of ChromA annotations

To compare ChromA against different algorithms, we used different metrics, the fraction of peaks covered, average peak coverage, and total coverage. We compute each metric from the intersection of bed files originating from the manually annotated regions versus algorithmically annotated regions. To compute the fraction of peaks covered (fpc), each manually annotated peak is intersected with the list of peaks algorithmically generated. If the intersection returns non-empty bases, the peak is considered intersected and recorded as such. The final metric value is computed by dividing the number of intersected peaks over the number of peaks $\left({\mathrm{fpc = }}\frac{{\# \, {\mathrm{of}}\,{\mathrm{intersected}}\,{\mathrm{peaks}}}}{{\# \, {\mathrm{manually}}\,{\mathrm{annotated}}\,{\mathrm{peaks}}}}\right)$. To compute the average peak coverage (apc), we again intersect each manually annotated peak and count the base pairs in the intersection over the total number of base pairs in the peak $\left({\mathrm{pc}} = \frac{{\# \, {\mathrm{bases}}\,{\mathrm{in}}\,{\mathrm{the}}\,{\mathrm{intersection}}}}{{\# \, {\mathrm{bases}}\,{\mathrm{in}}\,{\mathrm{the}}\,{\mathrm{manually}}\,{\mathrm{annotated}}\,{\mathrm{peak}}}}\right)$. The apc is computed as the mean of the pc for every manually annotated peak. We report the apc as mean +/− s.e.m. To compute the total coverage (tc), we add all the intersected bases and divide by the total number of bases in manually annotated peaks $\left({\mathrm{tc}} = \frac{{{\sum} {\# \, {\mathrm{bases}}\,{\mathrm{in}}\,{\mathrm{the}}\,{\mathrm{intersection}}} }}{{{\sum} {\# \, {\mathrm{bases}}\,{\mathrm{in}}\,{\mathrm{the}}\,{\mathrm{manually}}\,{\mathrm{annotated}}\,{\mathrm{peak}}} }}\right)$. The number of ChiP-seq events overlapping peaks serves as a recall metric. To compute algorithmic precision, we compute the number of peaks containing at least one ChIP-Seq event divided by the total number of peaks.

### DNAse-seq annotations

Data sets for DNAse-seq experiments for cell lines GM12878 and K562 were downloaded from the Encode project (wgEncodeUwDnaseGm12878AlnRep1.bam/Rep2.bam and wgEncodeUwDnaseK562AlnRep1.bam/Rep2.bam). For each corresponding cell line, we download ChIP-Seqs experiments from the encode project and merge them. For each binding event, we keep the center base-pair location and consider that a peak captures the binding event if it superimposed with this base-pair location. ChromA annotated peaks with option “dnase”. For Macs2, we use the following command: macs2 callpeak -t $file -f BAM -g mm -n $name -p 1e-2 -nomodel -shift -75 -extsize 150 -keep-dup all. For hotspot2, we first generate a reference for the hg19 genome by running extractcentersize.sh and then we annotate each file by using: hotspot2.sh -c $fchrom -C $fCenter -P -f 0.01 $file $outdir.

### ChromA model and core algorithm

Here, we present in more detail the entire ChromA’s generative process. The observed number of Tn5-binding events X_b_ at each base b is drawn independently through the process here described.

6 $$	{p_i \sim Beta(a_0,b_0)} \\ 	{\pi _i \sim Dir\left( {\alpha _0} \right)C} \\ 	{S_b \sim Mult(A_{S_{b - 1}})} \\ 	{X_b \sim Geo(X_b;p_{s_b})}$$

*S*_*b*_ denotes the chromatin state at base b, and it is distributed according to the transition matrix at the previous state. p is the probability of observing *X*_*b*_ number of binding events at base *b* given the current chromatin state. $a_0,b_0,\alpha _0$ are prior parameters. A denotes the HMM embedding of the HSMM and for a two-state model with 3 and 2 states it is written as:

7 $$\left( {\begin{array}{*{20}{c}} {p_{{\mathrm{Closed}}}} & {1 - p_{{\mathrm{Closed}}}} & {} & {} & {} \\ {} & {p_{{\mathrm{Closed}}}} & {} & {} & {} \\ {} & {} & {} & {} & {} \\ {} & {} & {1 - p_{{\mathrm{Closed}}}} & {} & {} \\ {} & {} & {p_{{\mathrm{Closed}}}} & {(1 - p_{{\mathrm{Closed}}})B(1;1,p_{{\mathrm{Open}}})} & {(1 - p_{{\mathrm{Closed}}})B(0;1,p_{{\mathrm{Open}}})} \\ {} & {} & {} & {p_{{\mathrm{Open}}}} & {1 - p_{{\mathrm{Open}}}} \\ {(1 - p_{{\mathrm{Open}}})B(2;2,p_{{\mathrm{Close}}})} & {(1 - p_{{\mathrm{Open}}})B(1;2,p_{{\mathrm{Close}}})} & {(1 - p_{{\mathrm{Open}}})B(0;2,p_{{\mathrm{Close}}})} & {} & {p_{{\mathrm{Open}}}} \end{array}} \right),$$

we used 5 and 2 as our fixed number of states, and although we perform computational experiments to fit p, these values were fixed at 1 × 10^−4^. *a*_0_ and *b*_0_ parametrize pseudocounts for the probability of observing a number of binding events in a particular base. We set these values to (1, 50) for the state that represents closed chromatin and (20, 10) for the state that represents open, however, the results are insensitive to these values. *α*_0_ denotes the prior pseudocounts for the initial state of the Markov process. Given our strategy that identifies batches surrounded by empty regions, we assume that the process starts in the closed state, *α*_0_ = (1000, 1). Again, the algorithm is insensitive to this value, as only the first few bases will be affected by it.

### ChromA single-cell data sets

To run ChromA on single-cell data sets, tsv files should be entered as input data, either as a raw file or as a tabix index file (this last file type is preferred for fast calculations). ChromA automatically builds an observation vector recognizing the type of input data by pooling single-cell information.

### Reporting summary

Further information on research design is available in the Nature Research Reporting Summary linked to this article.

## Supplementary information

Supplementary Information

Reporting Summary

## Data Availability

The accession number for the bulk ATAC-seq data reported in this paper is GSE113721. The accession number for the cut&run experiments is GSE104550. As reported above, single-cell data sets were downloaded from: https://support.10xgenomics.com/single-cell-atac/datasets/.

## Code availability

A Python implementation of ChromA is available for download on GitHub: http://github.com/marianogabitto/ChromA. Within the main page, detailed installation instructions are described. The website will be updated periodically with new versions.

## References

[1] Kornberg RD (1974). Chromatin structure: a repeating unit of histones and DNA. Science.

[2] Kornberg RD, Lorch Y (1992). Chromatin structure and transcription. Annu. Rev. Cell Biol..

[3] Zhang P, Torres K, Liu X, Liu CG, Pollock RE (2016). An overview of chromatin-regulating proteins in cells. Curr. Protein Pept. Sci..

[4] Smith ZD, Meissner A (2013). DNA methylation: roles in mammalian development. Nat. Rev. Genet..

[5] Mellor J (2005). The dynamics of chromatin remodeling at promoters. Mol. Cell..

[6] Mitchell PJ, Tjian R (1989). Transcriptional regulation in mammalian cells by sequence-specific DNA binding proteins. Science.

[7] Kohwi M, Doe CQ (2014). Temporal fate specification and neural progenitor competence during development. Nat. Rev. Neurosci..

[8] Slattery M (2014). Absence of a simple code: how transcription factors read the genome. Trends Biochem. Sci..

[9] Buenrostro J, Wu B, Chang H, Greenleaf W (2015). ATAC-seq: a method for assaying chromatin accessibility genome-wide. Curr. Protoc. Mol. Biol..

[10] Buenrostro JD (2015). Single-cell chromatin accessibility reveals principles of regulatory variation. Nature.

[11] Skene PJ, Henikoff JG, Henikoff S (2018). Targeted in situ genome-wide profiling with high efficiency for low cell numbers. Nat. Protoc..

[12] Kaya-Okur HS (2019). CUT&Tag for efficient epigenomic profiling of small samples and single cells. Nat. Commun..

[13] Luo C (2017). Single-cell methylomes identify neuronal subtypes and regulatory elements in mammalian cortex. Science.

[14] Inoue F (2017). A systematic comparison reveals substantial differences in chromosomal versus episomal encoding of enhancer activity. Genome Res..

[15] Lizio M (2017). Update of the FANTOM web resource: high resolution transcriptome of diverse cell types in mammals. Nucleic Acids Res..

[16] Johnson, M. J. & Willsky, A. S. Stochastic Variational inference for Bayesian time series models. in International Conference on Machine Learning (eds Xing, E. P. & Jebara, P.) 1854–1862 (PMLR, Bejing, China, 2014).

[17] Ciofani M (2012). A validated regulatory network for Th17 cell specification. Cell.

[18] Miraldi ER (2019). Leveraging chromatin accessibility for transcriptional regulatory network inference in T helper 17 cells. Genome Res..

[19] Durbin, R., Eddy, S. R., Krogh, A. & Mitchison, G. *Biological Sequence Analysis: Probabilistic Models of Proteins and Nucleic Acids* 1st edition. (Cambridge University Press, 1998).

[20] Guédon Y (2003). Estimating hidden semi-Markov chains from discrete sequences. J. Comput. Graph. Stat..

[21] Adey A (2010). Rapid, low-input, low-bias construction of shotgun fragment libraries by high-density in vitro transposition. Genome Biol..

[22] Creyghton, M. P. et al. Histone H3K27ac separates active from poised enhancers and predicts developmental state. *PNAS* **107**, 21931–21936 (2010). 10.1073/pnas.1016071107 PMC3003124 21106759

[23] McCarthy MT, O’Callaghan CA (2014). PeaKDEck: a kernel density estimator-based peak calling program for DNaseI-seq data. Bioinformatics.

[24] Feng J, Liu T, Qin B, Zhang Y, Liu XS (2012). Identifying ChIP-seq enrichment using MACS. Nat. Protoc..

[25] Tarbell ED, Liu T (2019). HMMRATAC: a hidden Markov ModeleR for ATAC-seq. Nucleic Acids Res..

[26] Li Q, Brown JB, Huang H, Bickel PJ (2011). Measuring reproducibility of high-throughput experiments. Ann. Appl. Stat..

[27] Ramirez F (2016). deepTools2: a next generation web server for deep-sequencing data analysis. Nucleic Acids Res..

[28] Nagano T (2013). Single-cell Hi-C reveals cell-to-cell variability in chromosome structure. Nature.

[29] Nagano T (2017). Cell-cycle dynamics of chromosomal organization at single-cell resolution. Nature.

[30] Canver MC (2018). Integrated design, execution, and analysis of arrayed and pooled CRISPR genome-editing experiments. Nat. Protoc..

[31] Liu Y (2019). Bisulfite-free direct detection of 5-methylcytosine and 5-hydroxymethylcytosine at base resolution. Nat. Biotechnol..

[32] Wang H, Mayhew D, Chen X, Johnston M, Mitra RD (2011). Calling cards enable multiplexed identification of the genomic targets of DNA-binding proteins. Genome Res..

[33] Henikoff JG, Belsky JA, Krassovsky K, MacAlpine DM, Henikoff S (2011). Epigenome characterization at single base-pair resolution. PNAS.

[34] Du Y, Murani E, Ponsuksili S, Wimmers K (2014). biomvRhsmm: genomic segmentation with hidden semi-Markov model. Biomed. Res. Int..

[35] Qin (2010). HPeak: an HMM-based algorithm for defining read-enriched regions in ChIP-Seq data. BMC Bioinform..

[36] Mammana A, Chung H (2015). Chromatin segmentation based on a probabilistic model for read counts explains a large portion of the epigenome. Genome Biol..

[37] Ernst J, Kellis M (2017). Chromatin-state discovery and genome annotation with ChromHMM. Nat. Protoc..

[38] Hoffman MM (2012). Unsupervised pattern discovery in human chromatin structure through genomic segmentation. Nat. Methods.

[39] Baker SM, Rogerson C, Hayes A, Sharrocks AD, Rattray M (2019). Classifying cells with Scasat, a single-cell ATAC-seq analysis tool. Nucleic Acids Res..

[40] Bravo Gonzalez-Blas (2019). cisTopic: cis-regulatory topic modeling on single-cell ATAC-seq data. Nat. Methods.

[41] Li et al. Identification of transcription factor binding sites using ATAC-seq. *Genome Biol.* **20**, 45 (2019). 10.1186/s13059-019-1642-2 PMC6391789 30808370

[42] Karabacak Calviello A, Hirsekorn A, Wurmus R, Yusuf D, Ohler U (2019). Reproducible inference of transcription factor footprints in ATAC-seq and DNase-seq datasets using protocol-specific bias modeling. Genome Biol..

