# Supplementary material for: Characterizing chromatin landscape from aggregate and single-cell genomic assays using flexible duration modeling
Source: Nat Commun. 2020 Feb 6;11:747. doi: 10.1038/s41467-020-14497-5 (PMC7004981; doi:10.1038/s41467-020-14497-5)
Supplement: Supplementary file 1 — Supplementary Information [file 41467_2020_14497_MOESM1_ESM.pdf]

Supplementary Information

Characterizing chromatin landscape from aggregate and single-cell genomic assays using flexible duration modeling

Gabitto et al.

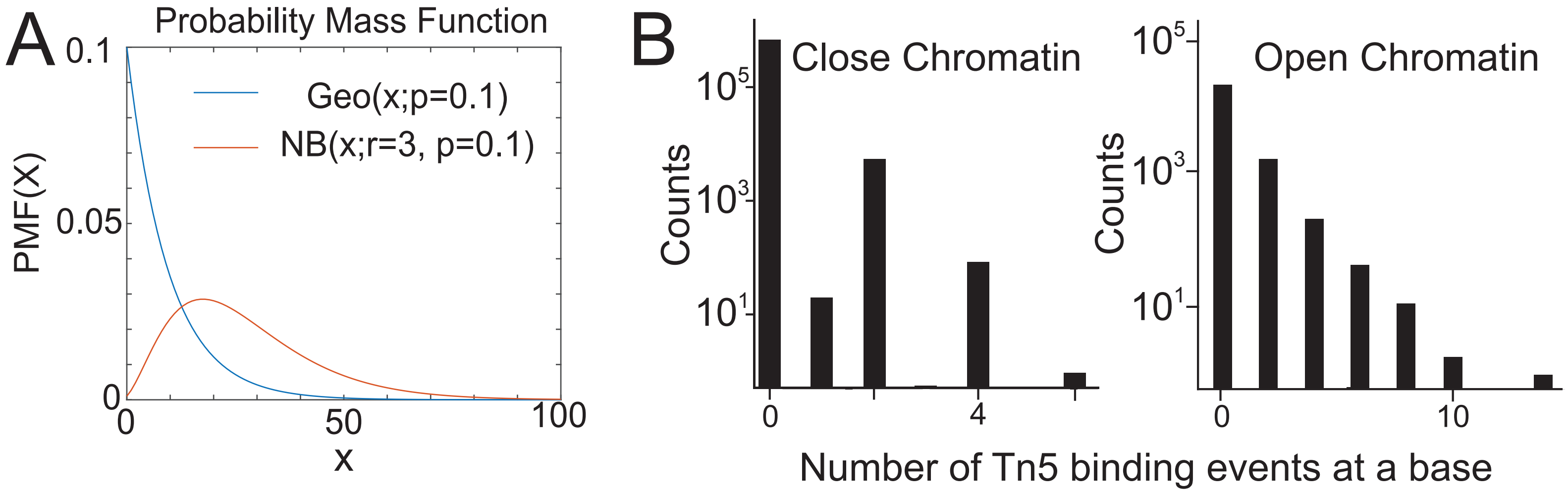

Supplementary Figure 1: Probability distributions characterizing Open and Closed Chromatin.

(a) Example of negative binomial distribution with parameters r=3, p=0.1 and geometric distribution with parameter p=0.1. The mode of the geometric distribution is always 0, which is not ideal to model genomic elements. (b) Histogram depicting the number of Tn5 binding events in manually annotated open and closed chromatin. Both distributions possess their maximum at 0 and can be effectively described with a geometric distribution.

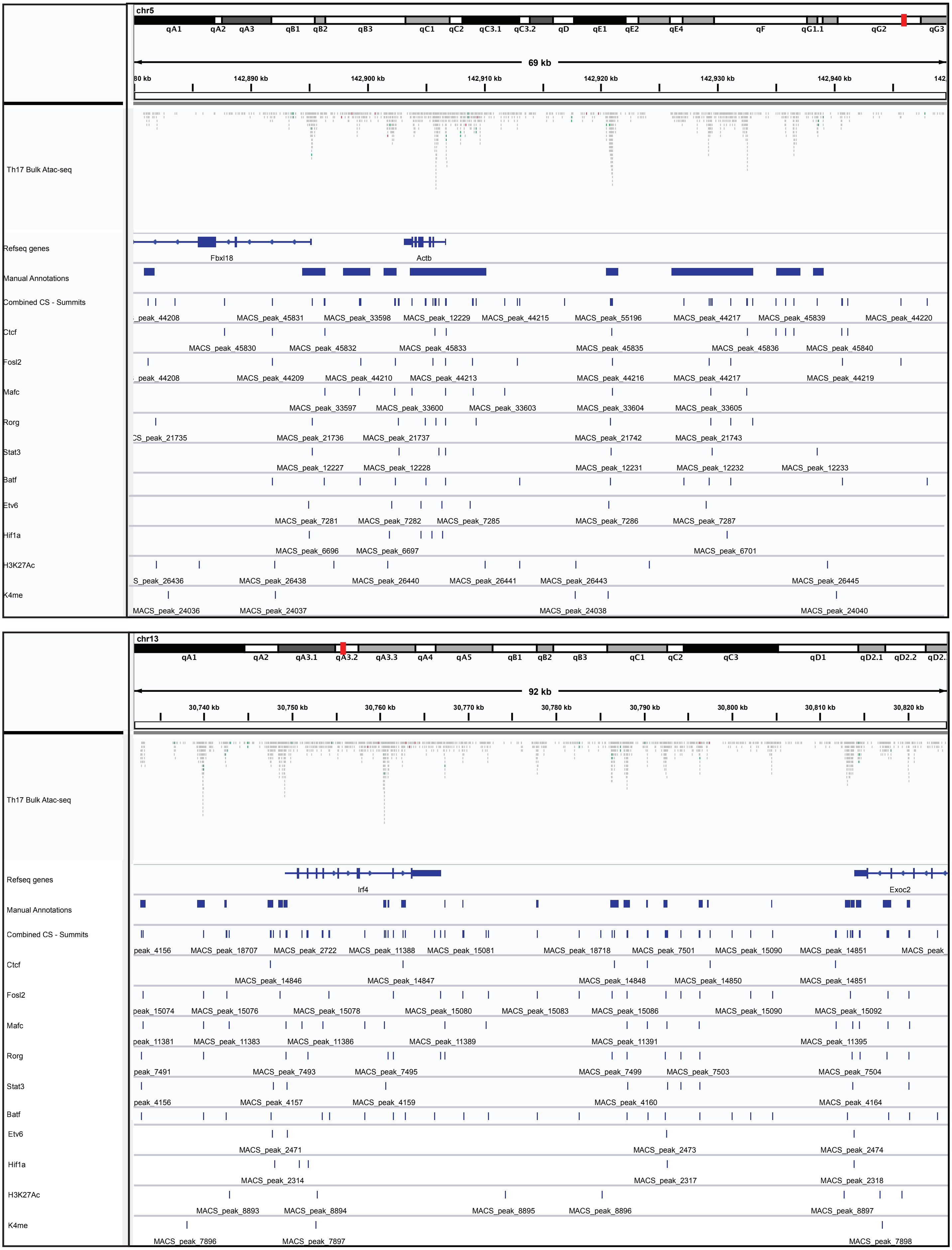

Supplementary Figure 2: Example Manual Annotations on Selected Genomic Regions.
Manual annotations on two out of ten selected genomic regions. ChIP-seq binding locations collected for different transcription factors are used to delineated chromatin accessible regions.

A

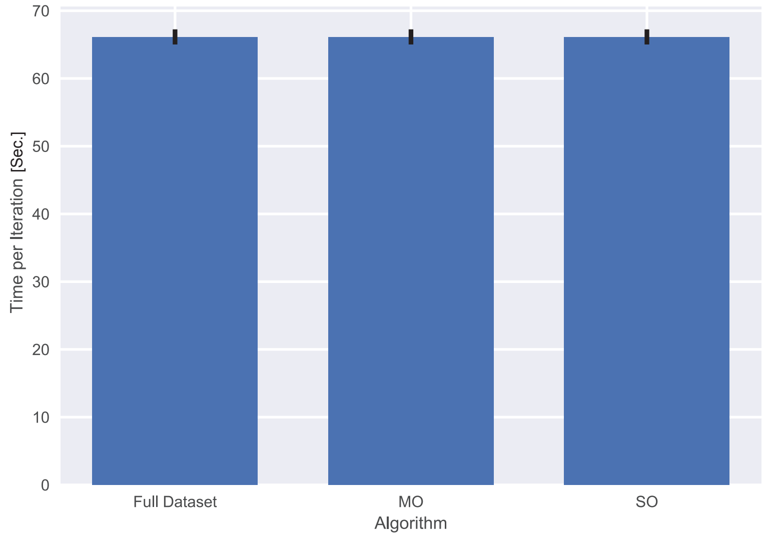

B

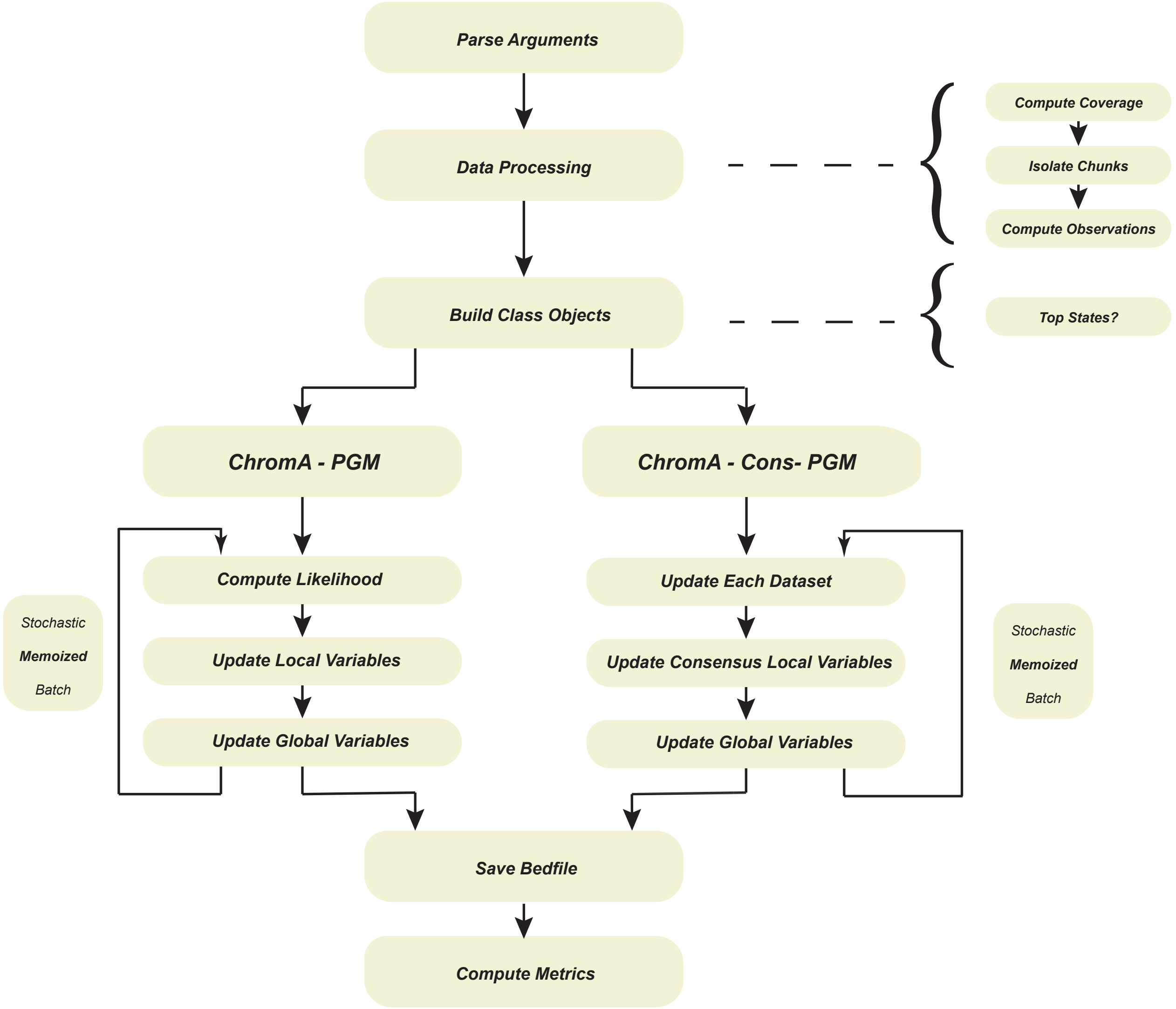

Supplementary Figure 3: ChromA Running Time and Complete Computational Pipeline.
(a) ChromA running time of one iteration on mouse chromosome 1 (mean +/- sem). Minimal computational overhead is observed when using batch algorithms. Full refers to our algorithm that uses no batches. MO: memoized optimization and SO: stochastic optimization. Scale in seconds.
(b) ChromA computational pipeline is illustrated.

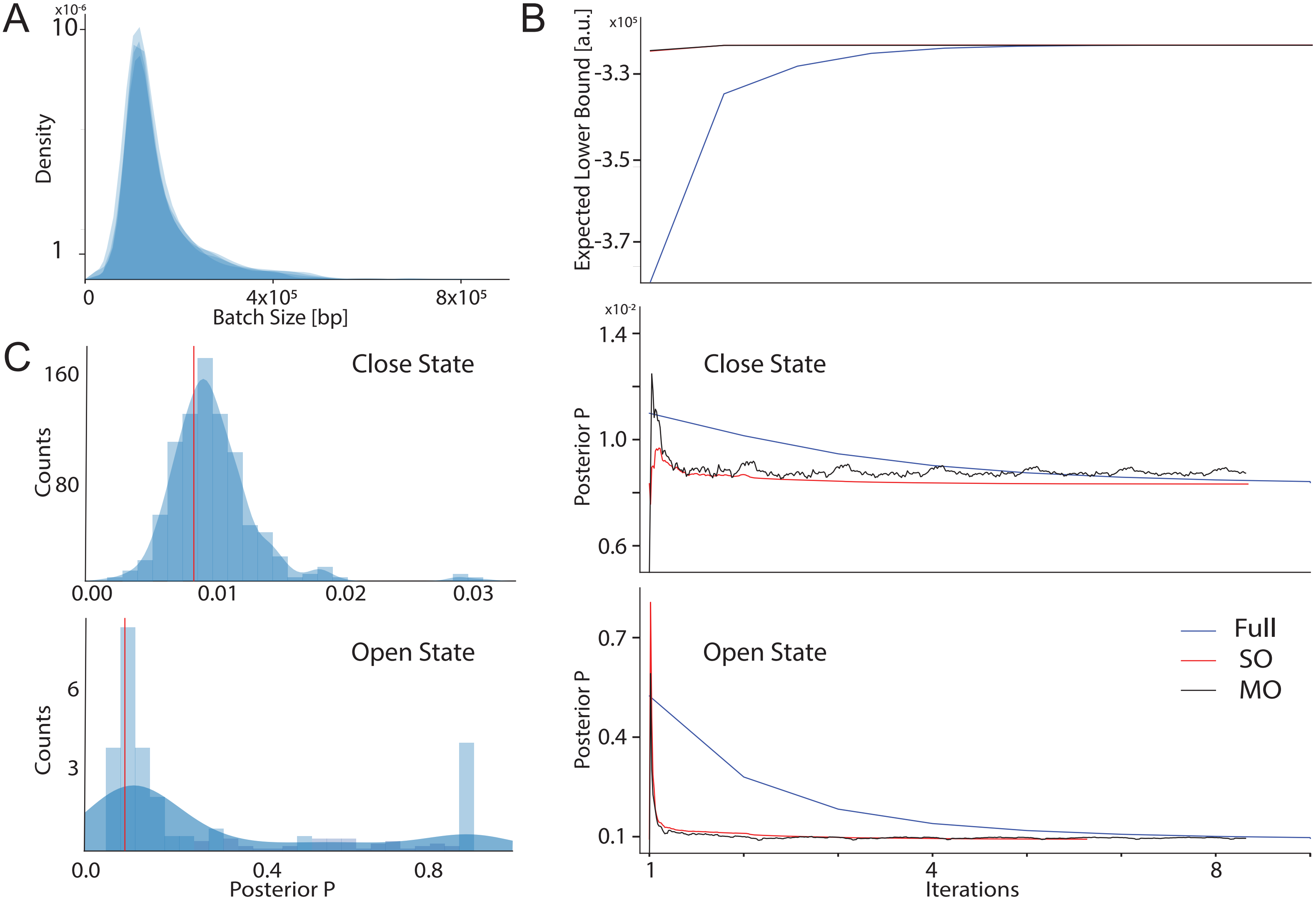

Supplementary Figure 4: Scaling of ChromA Parameters' Inference.

Parameter inference in ChromA (performed for each batch of data or set of replicate datasets) is accelerated by distributing computational load in batches.

(a) Chromosomal regions are divided into batches of data by identifying flanking regions lacking a significant number of reads. These batches of data have a minimum length of 100 kbp and experimentally determine maximum of less than 600kbp.

(b) To ensure computational scalability, we compare parameter inference with no batch acceleration (Full) against stochastic (SO) and memoized optimization (MO). Algorithms run for 10 iterations on a dataset composed of chromosome 19 of Th17 cells using our two-state bulk inference algorithm. MO and SO algorithms converge faster when compared to the Full algorithm, seeing as a faster convergence of the expected lower bound and the open and closed chromatin states' parameters.

(c) Entire chromosomes can be annotated by using open and closed states' parameters that generalize chromosome wide. Histogram depicting open and closed probability parameters fit independently in each data batch. On red is the posterior parameter value when using a single set of open and closed parameters chromosome wide.

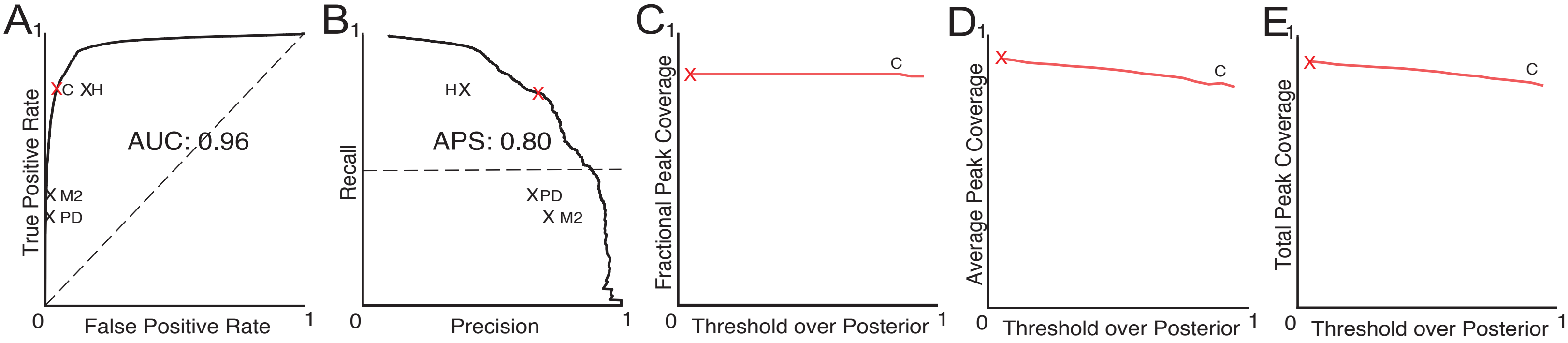

Supplementary Figure 5: Evaluating Threshold Selection on Manually Annotated Regions
(a, b) Base-pair level Receiver Operating and Precision Recall curves, respectively. ChromA recalls a greater correct number of manually annotated bases while controlling false positive rate and retaining precision.
(c, d, e) Peak level metrics computed for different threshold values (ranging from 0.05 to 0.95).
(c) The fraction of peaks recovered in our manually annotated regions remains unaltered in a wide range of threshold values (0.05 – 0.9).
(d), (e) As threshold is increased, the fraction of each peak covered and the total coverage per manually annotated region decreases in a range of 0.9 to 0.8.
Red cross indicates Chroma's results with threshold of 0.5. C, M2, H and PD denote results for algorithms ChromA, MACS2, HMMRAtac and PeaKDEck.

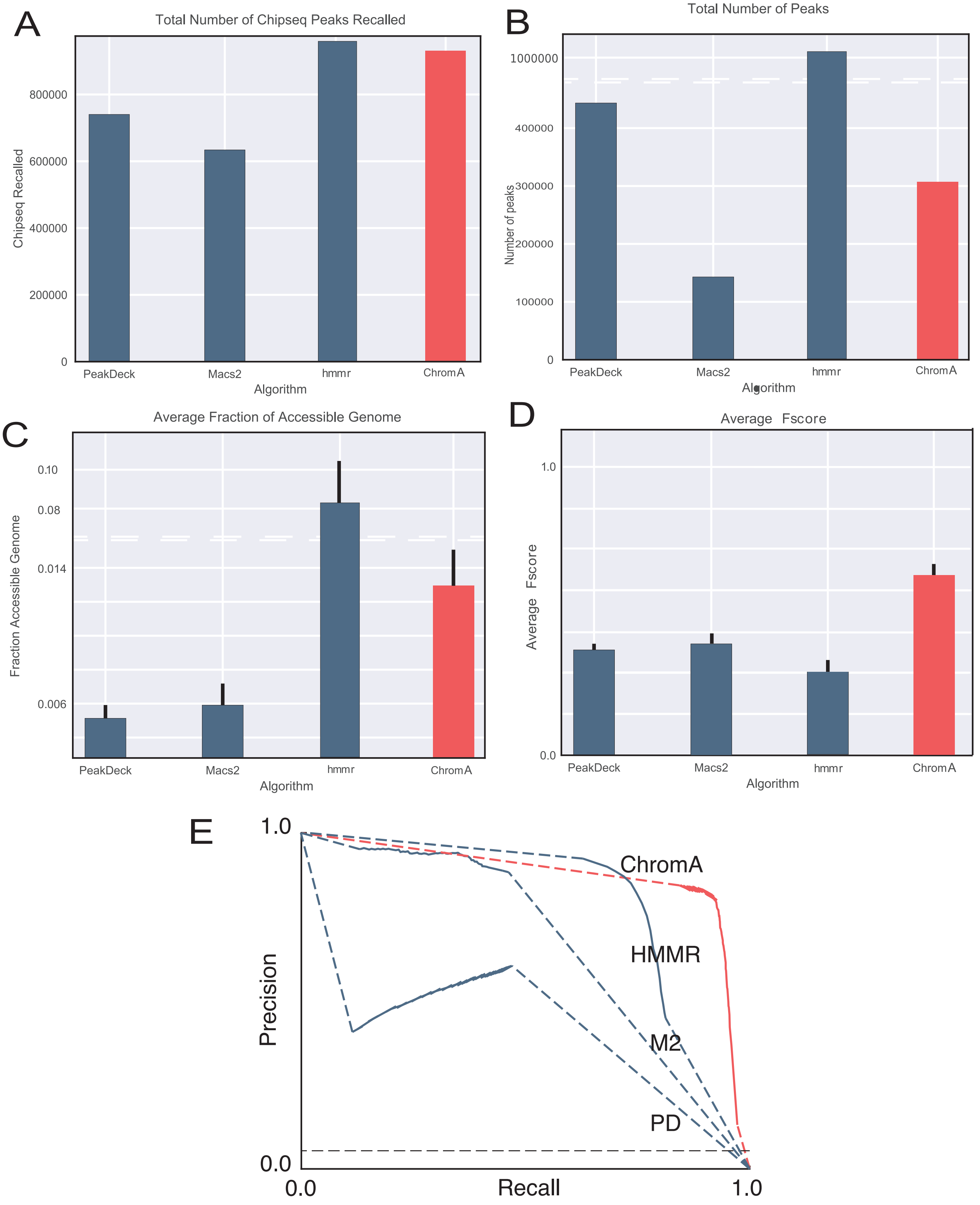

Supplementary Figure 6: Extended Genome-wide Validation of ChromA Algorithm.
Validation of genome-wide annotations performed in 4 additional datasets of sorted and CD4+ treated after 48hr Th17 cells.
(a) Number of ChIP-seq peaks recalled
(b) Total number of peaks
(c) Average fraction of the genome annotated as accessible.
(d) Average F1-score computed on the 4 extended validation datasets.
(e) Precision recall curve for dataset used in Figure 3 of the main text.

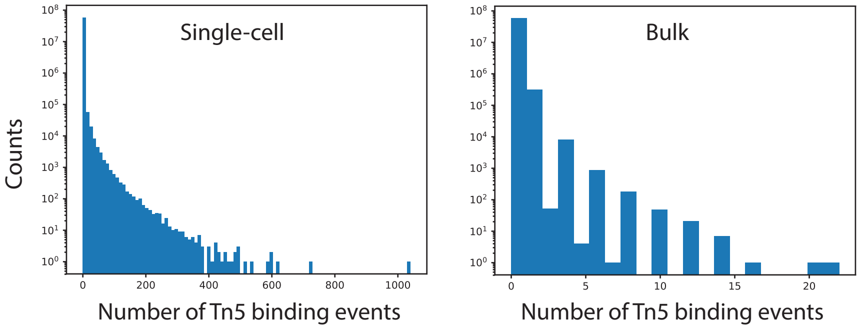

Supplementary Figure 7: Single-cell Data Sets Exhibit Extended Dynamic Range Compared to Bulk Data Sets.
Histogram of Tn5 binding events in single-cell data set of 10000 GM12878 human cells (left) and bulk Th17 mouse cells (right). Counts are depicted in log scale. Due to high level of sparsity, the number of reads in bulk dataset do not typically surpasses more than 50 Tn5 binding events per base. This is in contrast to single cell datasets in which the number of Tn5 binding events can reach on the order of thousands.

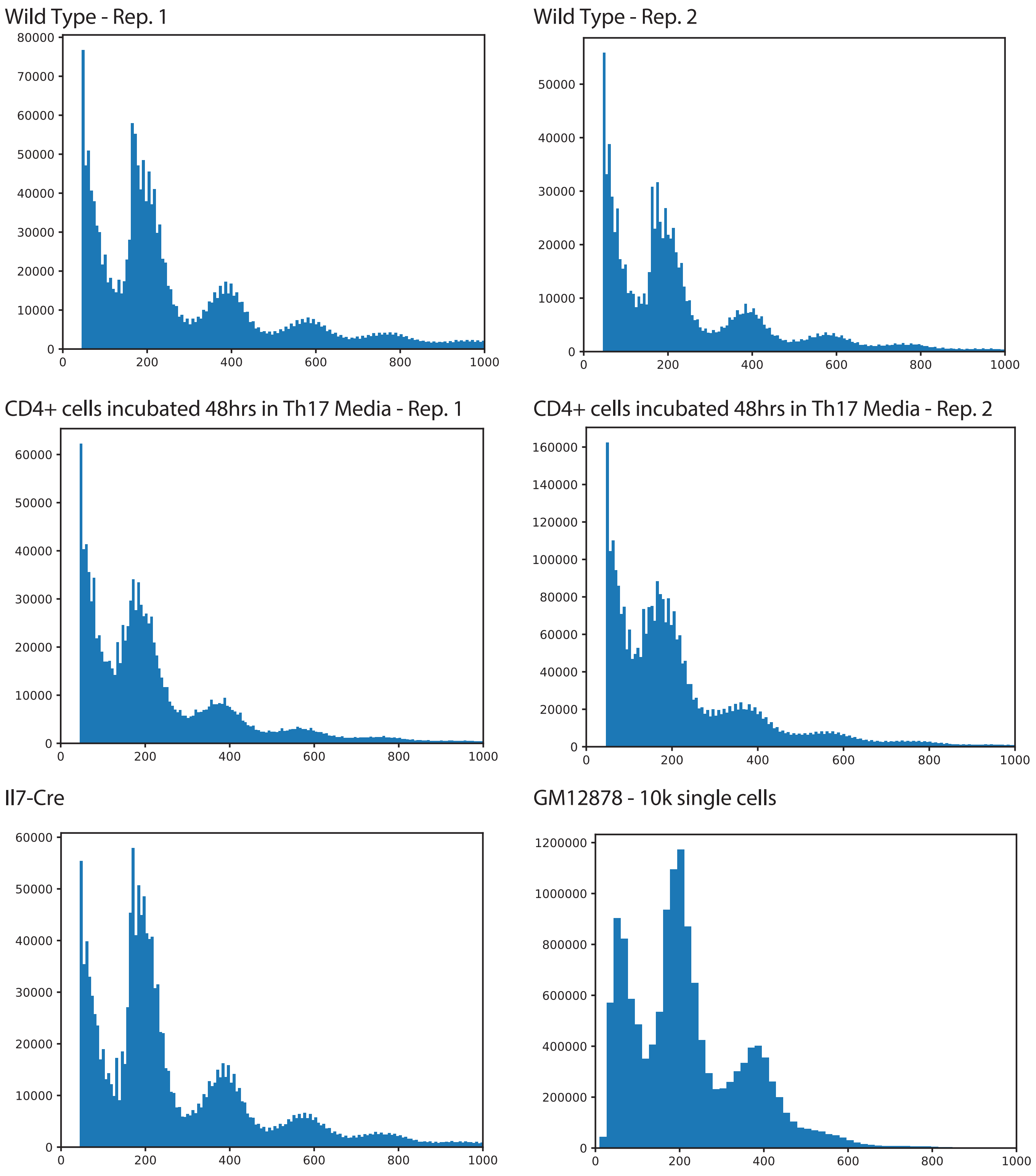

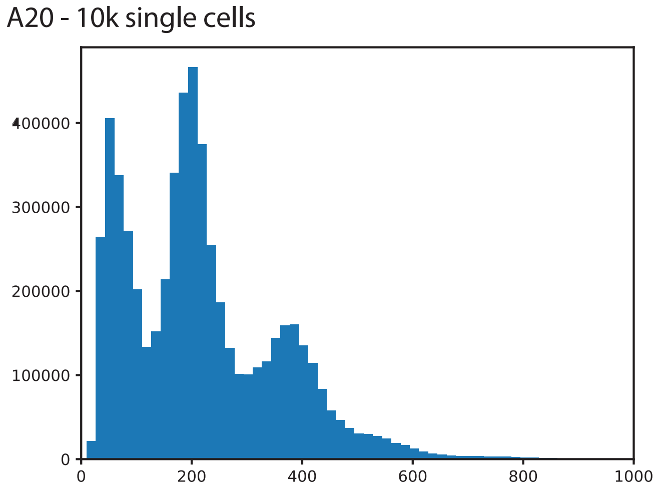

Supplementary Figure 8: Insert Size Distribution for Bulk and Single Cell Datasets.
Histogram depicting insert size distribution computed for every dataset used in this work. In every plot, first peak corresponds to nucleosome-free regions and subsequent peaks correspond to mono, bi and tri nucleosome reads.

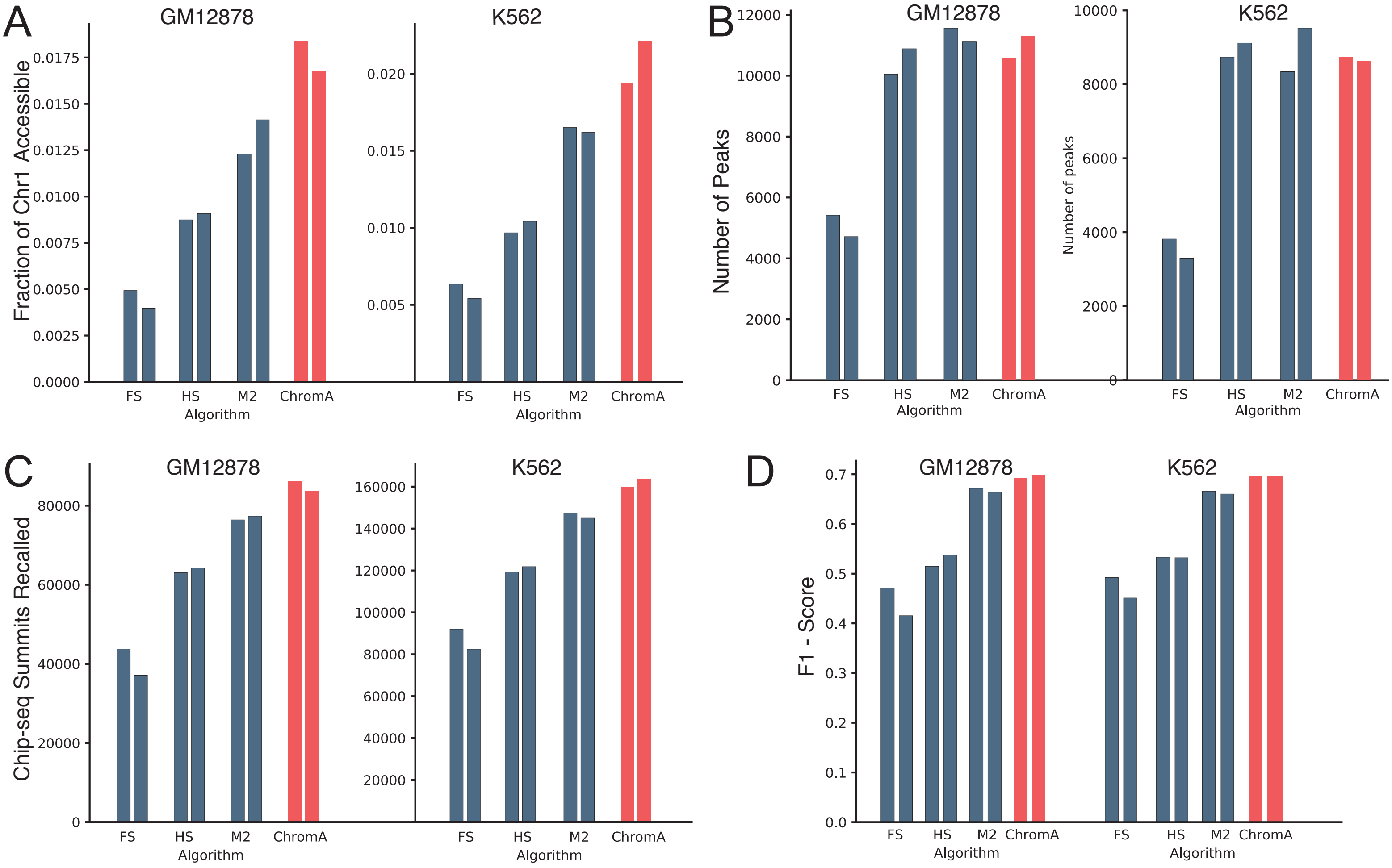

Supplementary Figure 9: ChromA effectively identifies accessible chromatin from DNAse-seq experiments. ChromA effective genome-wide performance recalls the highest number of ChIP-seq calls maintaining a comparable number of peaks. For each algorithm, we annotate two technical replicates datasets for human GM12878 and K562 cell lines. In all plots, ChromA indicates our newly developed ChromA algorithm, M2 indicates macs2, FS indicates F-Seq algorithm and HS indicates HotSpot2 algorithm.

(a) Fraction of Chr1 annotated as accessible.

(b) Total number of peaks annotated.

(c) Number of ChIP-seq binding events recalled.

(d) F1-score.

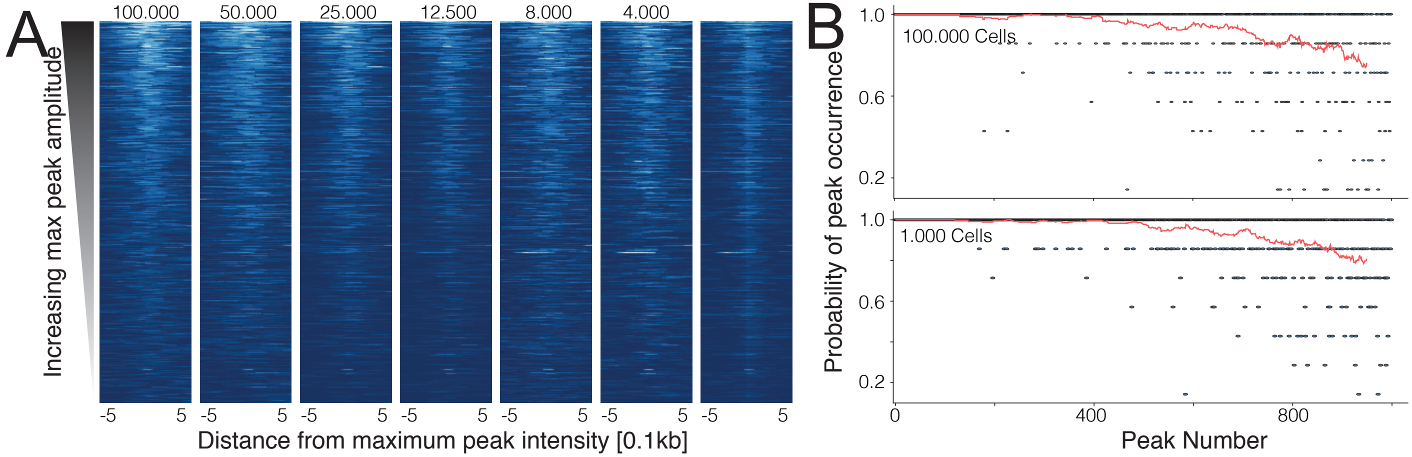

Supplementary Figure 10: ChromA identifies TF binding events from cut&run experiments.
(a) Annotated regions identified in the 100k cells Cut&Run dataset are sorted by their aggregated number of reads per region. This is done after down-sampling the dataset to 50, 25, 12.5, 8, 4, and 1 thousand cells and running ChromA on the subset of cells selected. The annotated regions for each dataset (the full and down-sampled datasets) are displayed centered on the maximum for each region.
(b) Top. Annotated regions on the dataset consisting of 100k cells are sorted according to their aggregated number of reads. Then, for each region, we compute the probability of observing that peak in all the different subsets described above. Bottom, we repeat the previous analysis starting from regions identified on the 1k cells dataset. This test demonstrates that the most significant features detected in the Cut&Run dataset are reliably detected by Chroma even after extreme down sampling, an important behavior for the analysis of single cell data and explorations of cell heterogeneity.

Supplementary Table 1

Description	Dataset Type	FRIP	SNR	# Reads	Extrapolated #Reads	Insert Size Metric
Mouse, Wild type TH17 sorted cells Replicate 1	Bulk	0.35	7.5	20.86e6	2e7	3.1
Mouse, Wild type TH17 sorted cells Replicate 2	Bulk	0.32	7.6	11.78e6	1e7	2.4
Mouse, CD4+ sorted cells Incubated in TH17 media for 48 hrs. Replicate 1	Bulk	0.13	4.1	15.39e6	2e7	2.4
Mouse, CD4+ sorted cells Incubated in TH17 media for 48 hrs. Replicate 2	Bulk	0.06	3.8	38.80e6	4e7	2.2
Mouse, Il7 - cre sorted cells Replicate 1	Bulk	0.22	4.4	18.36e6	2e7	4.2
Human, GM12878 10k - 500 cells.	Single-cell	0.45 - 0.65	4.0 - 4.8	-	8e6 - 2e8	3.7 - 4.2
Mouse, A20 10k - 500 cells.	Single-cell	0.41 - 0.52	6.1 - 7.1	-	6e6 - 8e7	3.5 - 3.7

Supplementary Note 1 - User Guide.

1. **Installation.**

ChromA can be installed by running:

pip install git+https://github.com/marianogabitto/ChromA

2. **Initial Checkup.**

Initial tests on the correct installation and correct set up of the system can be run by doing:

ChromA -v

3. **Example of running ChromA – Single File.**

```
chroma -i "file.bam" --species human  -sb output.bed
```

4. **Example of running consensus ChromA.**

```
chroma -i "file1.tsv" "file2.bam" --species human  -sb output12.bed
```

5. **Chrom – Complete List of Options.**

-i, --input: One or more input files to be processed. Files must have extension bam or tsv.
-th17, --regionsTh17: Parse reads and run ChromA only on selected Th17 regions (Optional, default = *False*)
-it, --iterations: Number of iterations to fit the algorithm (Optional, default = *10*)

-spec, --species: Genome Species. Options: mouse, human (Optional, default = *mouse*)
-inf, --inference: Inference algorithm. Options: batch, mo, so (Optional, default = mo)
-bl, --blacklisted: Remove Blacklisted peaks. (Optional, default = True)
-sb, --saveBedFile: Name of bedfile to save the data. To avoid saving use "False". Required, default = "bed_file")
-sp, --savePickleFile:default='False', help='save pickle file (default is False)')
-v, --version: report version and stop execution.

Supplementary Note 2 - **Scaling Chromatin Annotations Genome-wide.**

ChromA relies on approximate algorithms to perform inference on its parameters. To perform scalable inference in ChromA we use variational inference [1]. Further acceleration can be achieved by exploiting variational techniques that update parameters using batches of data [2, 3] (in our case, splitting the genome into chromosome segments). However, the Markovian interdependency of the chromatin state at each base pair limits the appropriateness of applying such methods, as breaking the continuity of the dataset would break the dependency of each base pair on the past state. To resolve this impediment, previous methods have used different techniques to accommodate these boundary effects [4], the simplest heuristic approach being the addition of buffers at each side of a batch (where each batch is a contiguous genomic region). The computational complexity of parameter inference in our algorithms scales with the length of the datasets. Applying buffering techniques results in additional computation time, an undesired effect when working with entire genomes. An ideal algorithm would be able to perform inference in batches and at the same time would retain calculations on the order of the length of the genome.

Considering both these boundary problems and the relevant biology, we developed a simple approach for addressing this issue. We identify *empty regions* in which no Tn5 binding events occurred in a window of 100 bp (reasoning that closed regions of sufficient size erase any memory effect due to the finite memory Markovian process and assume that the chromatin state in these regions can be regarded as closed). We define a batch as a region flanked by empty regions. Although batches defined in this manner are of variable size, we aim at batch sizes of 100 kbp and observe on real data the successful partition of chromosomes (Supplementary Figure 7a). This procedure permits us to compare batch-based inference algorithms. These algorithms run with minimum or no apparent computational overhead (Supplementary Figure 8a). We explored different inference algorithms, Memoized Online Variational Inference (MO) being our preferred method here. MO permits acceleration comparable to stochastic methods, ensures scalability, and provides

exact inference at the end of each iteration (Supplementary Figure 7b). The introduction of parameter inference in batches with biologically sound, data-driven, boundaries also allows us to consider how emission parameters characterizing chromatin accessibility vary in different chromosomal regions. To study parameter variability in different chromosomal regions, we fit Chrom including a set of open and closed parameters in each batch. From this experiment, we observed that a single set of parameters generalizes well to most chromosomal regions (Supplementary Figure 7C). In summary, the presented algorithmic acceleration techniques permit us to create a fast, scalable algorithm to annotate chromatin accessibility of entire genomes and allow integration of information from entire chromosomes. For completeness, we include the entire processing pipeline in supplemental Supplementary Figure 8b.

Supplementary References

- 1. Jordan M.I., Ghahramani Z., Jaakkola T. S., Saul L.K. An Introduction to Variational Methods for Graphical Models. *Machine Learning*, **37**, 183-233 (1999).
- 2. Hughes, M. C and Sudderth, E. Memoized Online Variational Inference for Dirichlet Process Mixture Models. *Advances in Neural Information Processing Systems* **26**, 1133--1141 (2013).
- 3. Hoffman M.D., Blei D.M., Wang C. Stochastic Variational Inference. *Journal of Machine Learning Research* **14**, 1303-1347 (2013).
- 4. Foti, N., Xu, J., Laird D., Fox, E. Stochastic variational inference for hidden Markov models. *Advances in Neural Information Processing Systems* **27**, 3599-3607 (2014).
